# Supplementary material for: Transient beta activity and cortico-muscular connectivity during sustained motor behaviour
Source: Prog Neurobiol. 2022 Jul;214:102281. doi: 10.1016/j.pneurobio.2022.102281 (PMC9742854; doi:10.1016/j.pneurobio.2022.102281)
Supplement: Supplementary file 1 — Supplementary material. [file mmc1.docx]

**Supplementary Figures**


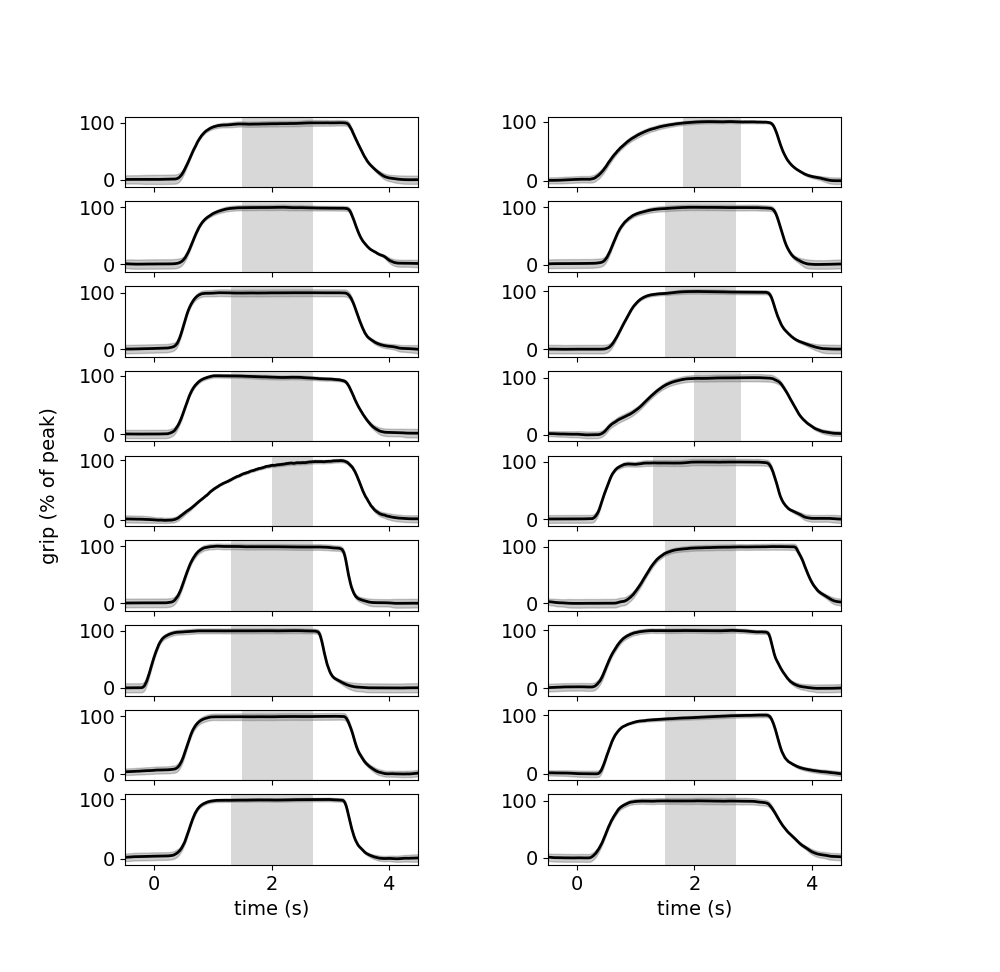


**Figure S1. Across-trial average of gripper signal in single participants.** The shaded areas around the black lines represent 95% confidence intervals. The shaded rectangular shaded area depicts the central part of the temporal window of interest during which beta ON and OFF periods were identified.


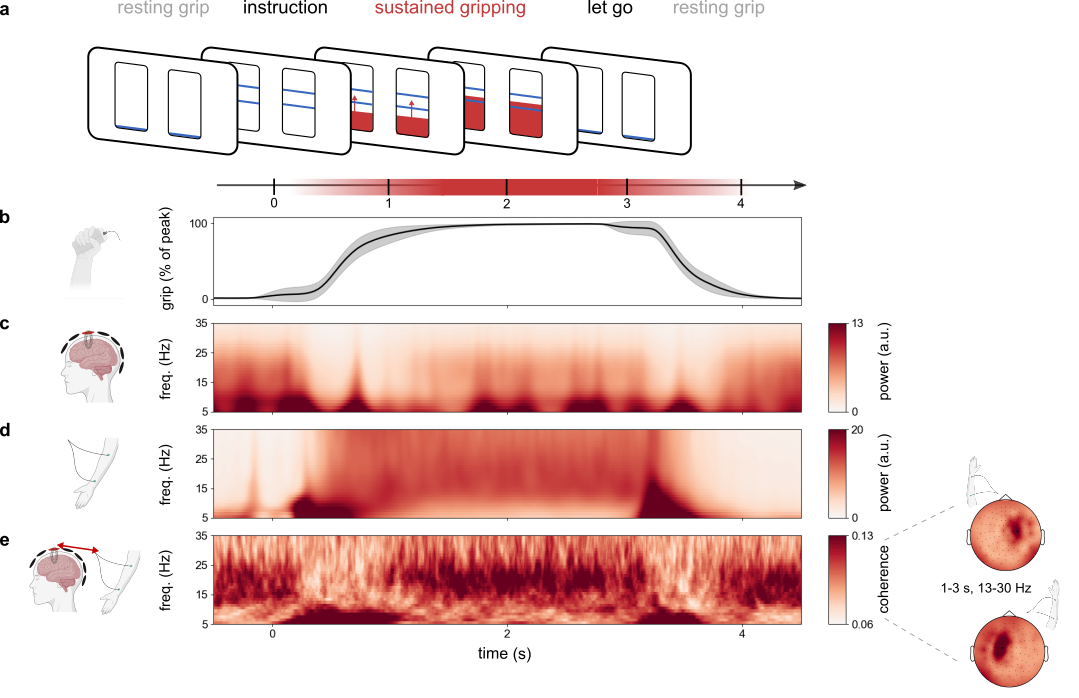


**Figure S2.  *Trial-average beta activity and connectivity appear sustained during sustained motor behaviour (gripping) across participants.* a**) Schematic of a single trial. Before each trial, participants held the gripping devices in both hands (resting grip). At time 0, two horizontal lines, indicating the gripping strength, appeared on the bars on the screen, prompting participants to grip. Participants began gripping until they reached a steady grip at the indicated strength, which they sustained for ~2s. The drop of the horizontal lines to the bottom of the bars indicated the end of a trial and the return to resting grip. **b**) Average grip output across trials and across left and right gripping devices across participants, expressed as a percentage of the maximum force. Percentage of maximum grip output was defined relative to each trial. Shading indicates 95% confidence intervals. **c**) Time-frequency spectrum of trial-average activity in selected motor MEG channels across participants. Selected MEG channels correspond to those with maximal cortico-muscular coherence during sustained gripping (see methods) and they lie over the left and right motor cortices, as shown in the topographical distribution in **Figure S2e**. **d**) Time-frequency spectrum showing average EMG activity across both forearms across participants. **e**) Time-frequency spectrum of cortico-muscular coherence (phase coupling) between the selected MEG channels and the contralateral forearm muscles. Topographies show coherence with the left and right forearms, averaged over the indicated time-frequency window across participants.


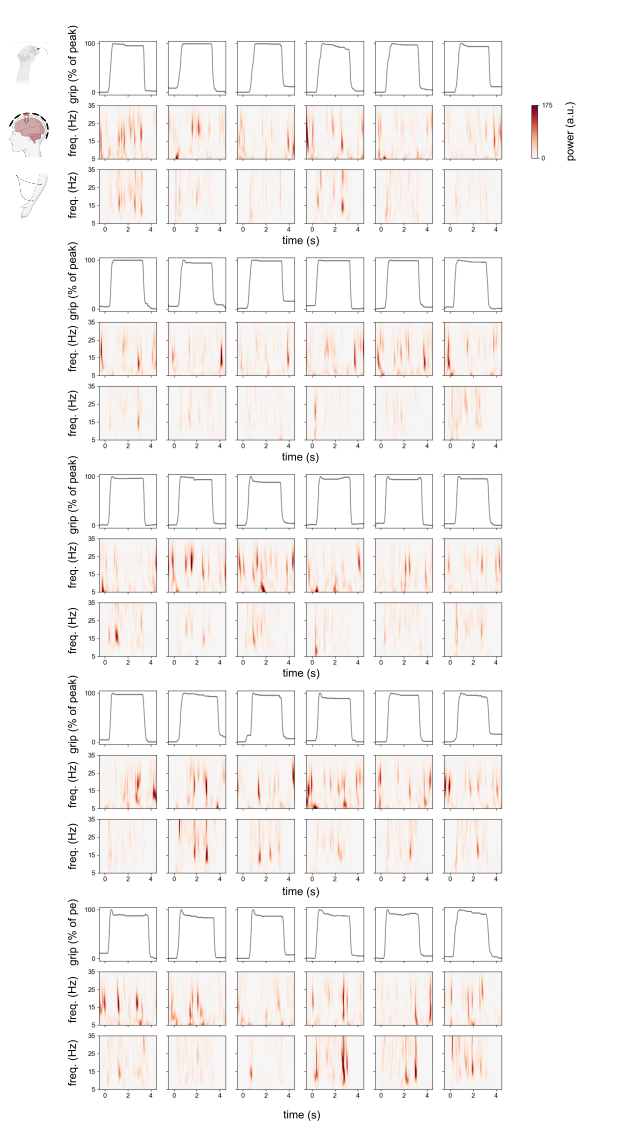


**Figure S3. Single trials in the brain, muscle and gripper.** Gripper traces together with time-frequency spectra in brain and muscle in 30 example trials from the same participant whose trial-average data is shown in **Figure 2**.


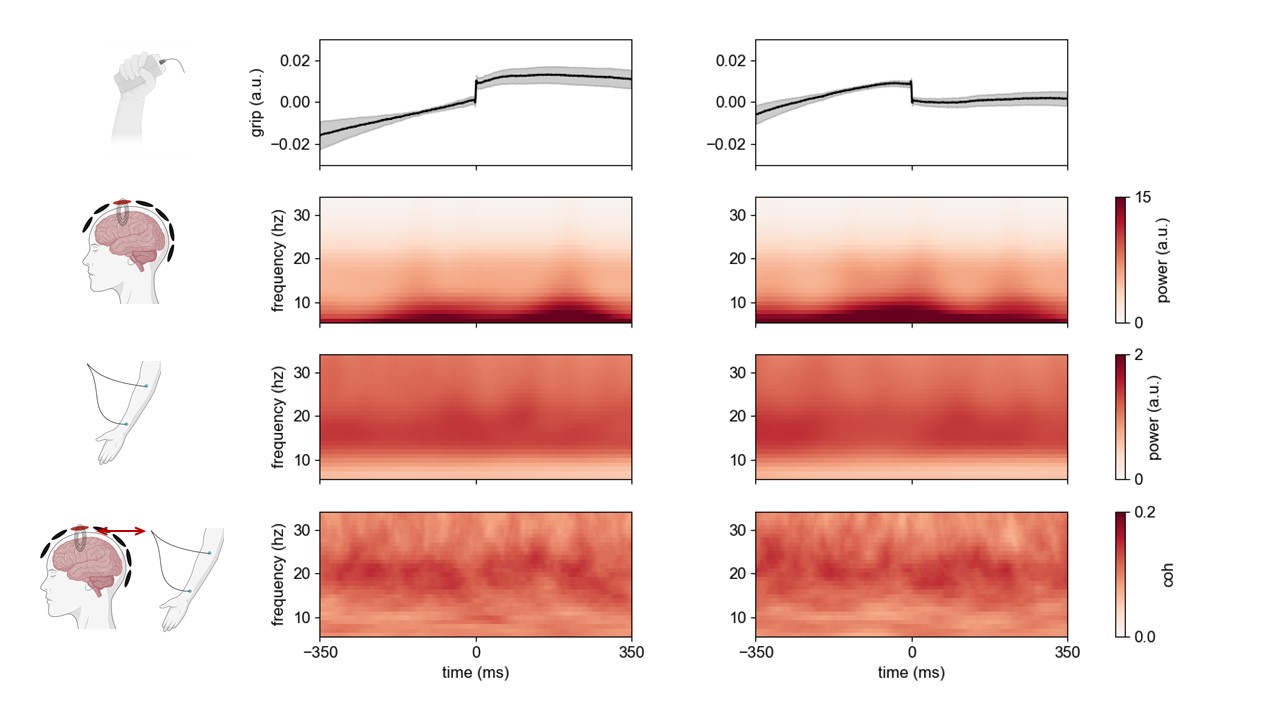


**Figure S4. Data aligned to periods of small changes in grip revealed no systematic increases in beta power nor CMC around minor grip adjustments.** We calculated the maximum and minimum points of the differential of the grip signal during the period of isometric contraction per trial. MEG, EMG and coherence data were aligned to such upwards and downwards grip adjustments separately across all trials and all participants (N=18).


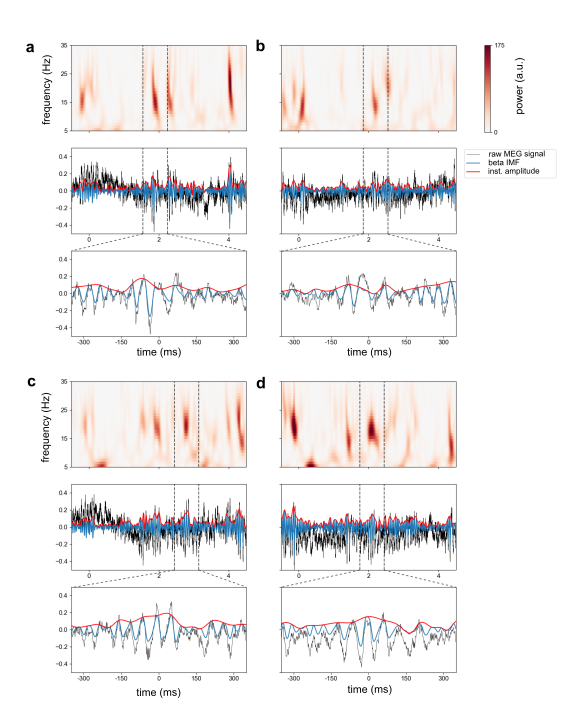


**Figure S5. Raw MEG signal, beta IMF and instantaneous amplitude of beta IMF during trials shown in Figure 2.** a-d single trials shown in Figure 2. Top: wavelet-convolved time-frequency spectra during the trial. Middle: raw MEG trace (black), beta IMF of the MEG signal (blue), and instantaneous amplitude of beta IMF (red) for the duration of the trial. Dotted lines depict representative individual beta events. Bottom: zoomed in version of the individual beta events.


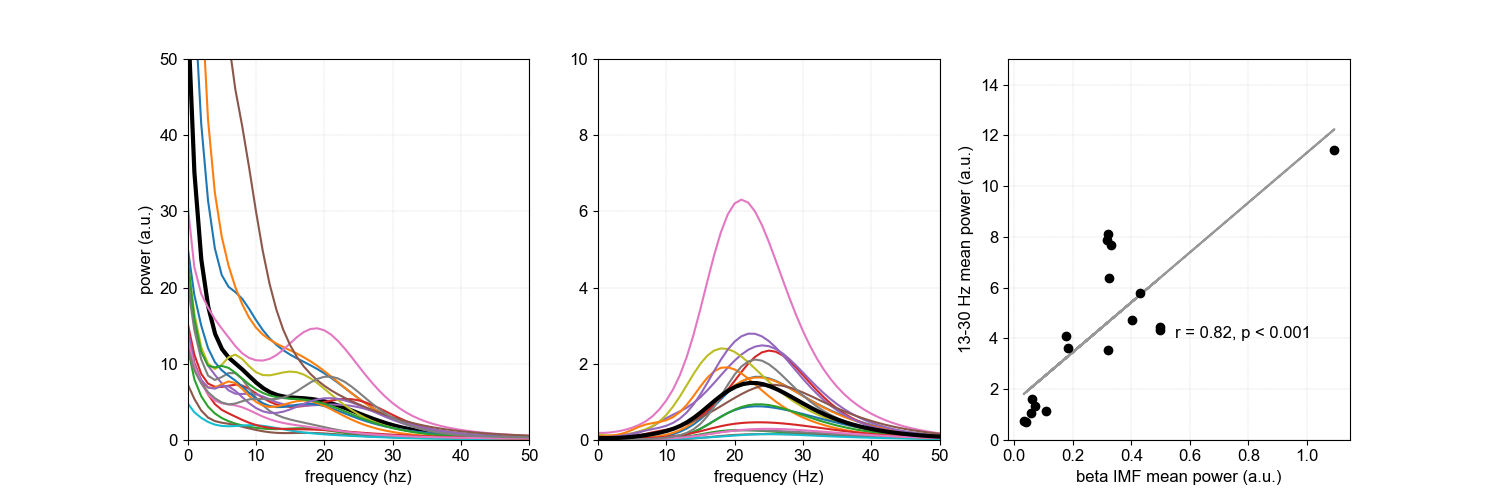


**Figure S6. Beta IMF captures beta component and isolates it from other frequency bands.** Left: power spectra for each participant (N = 18) and across-participant mean as black line. Middle: power spectra of beta IMF for each participant and average in black. Right: correlation between total power of beta IMF and average power in 13-30Hz frequency range (r = 0.82, p < 0.001).


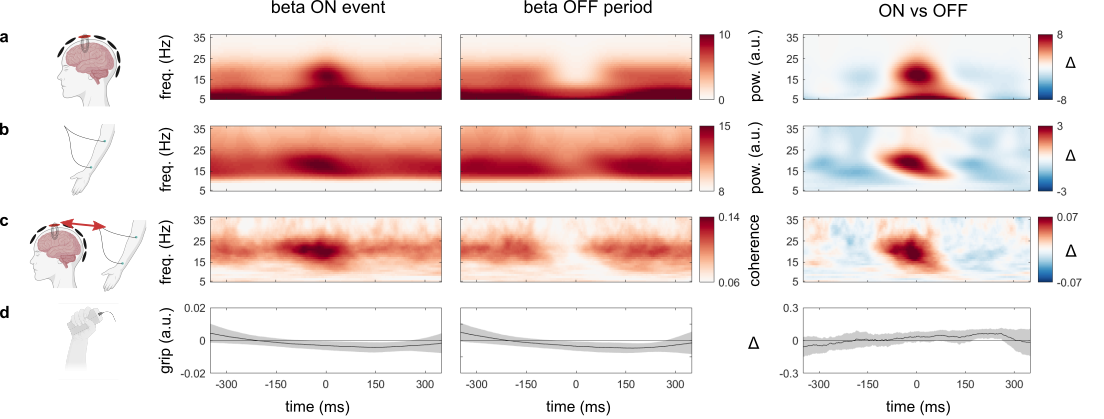


**Figure S7. Data aligned to beta ON and OFF periods (identified using a simple median power threshold) in the brain reveal transient beta in brain, muscle, and connectivity despite sustained motor output.** Beta ON events were identified as periods of beta power (13-30 Hz) higher than the median beta power per trial in the wavelet-convolved data from the two motor MEG channels during the ~1-3s period of sustained gripping (see methods). Beta OFF periods were periods with beta power below the median. After ON and OFF period detection, data were aligned and averaged across all trials and all participants (N=18). Columns from left to right represent our four signals of interest (**a-d**) aligned to the central point of a beta ON event (left), a beta OFF period (middle) and the difference between them (right). **a**) MEG time-frequency spectrum, **b**) EMG time-frequency spectrum, **c**) CMC time-frequency spectrum and **d**) gripper signal. Shading indicates 95% confidence intervals. Back outlines in the right time-frequency maps indicate significant clusters.


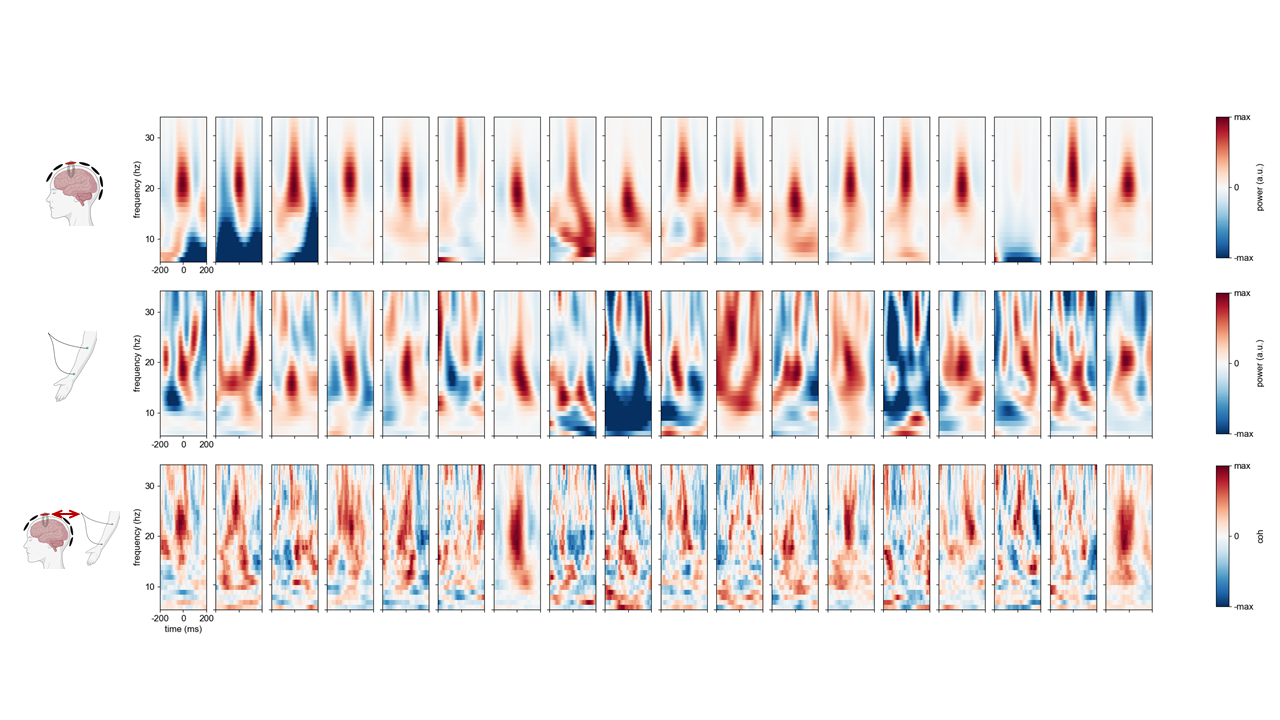


**Figure S8. Difference between data aligned to beta ON and OFF periods in the brain, the muscle, and their connectivity.** Beta ON events and OFF periods were identified using Empirical Mode Decomposition (EMD) in the selected MEG channels during the ~1-3s period of interest. After ON and OFF period detection, data were aligned and averaged across all trials per participant (N=18). The top row is the difference between beta ON events and OFF periods in the MEG signal, the middle row in the EMG signal and the bottom row in the CMC.


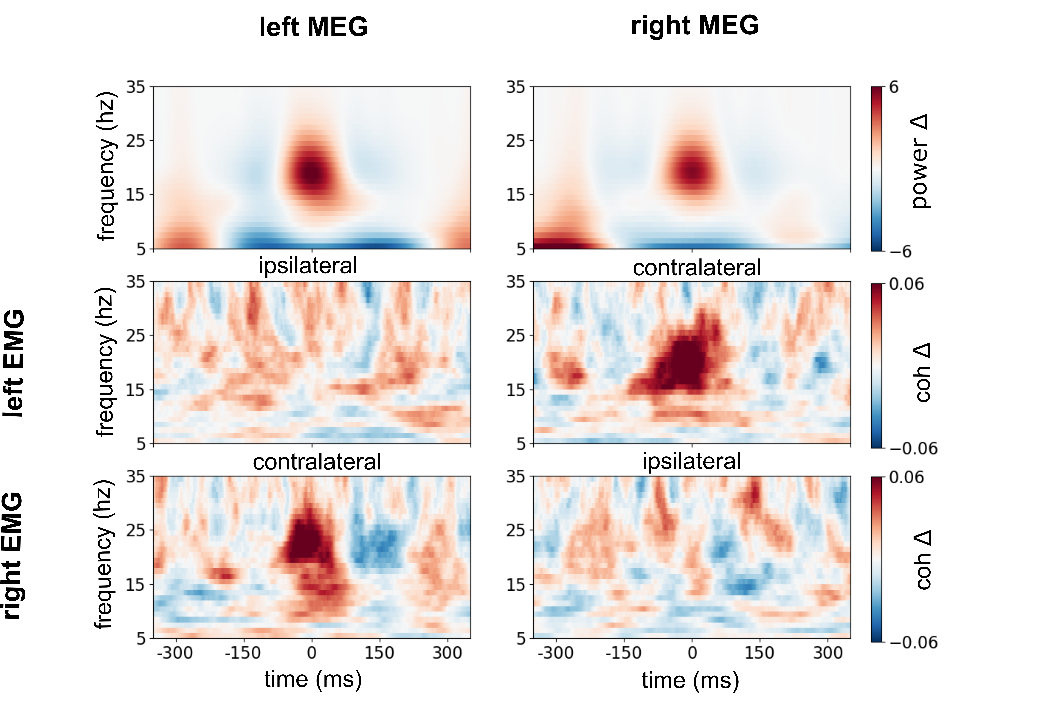


**Figure S9. Beta ON vs OFF period time-frequency spectra and CMC difference in the left and right MEG channels.** Beta ON events and OFF periods were identified using Empirical Mode Decomposition (EMD) in the selected MEG channels during the ~1-3s period of sustained gripping. ON and OFF beta periods were identified in the left and right MEG channels independently. We calculated CMC with the ipsilateral and contralateral EMG signals at the time of beta ON and OFF events in left and right MEG channels. We calculated the difference in MEG time-frequency spectra and CMC between ON and OFF periods in left and right MEG channels independently.


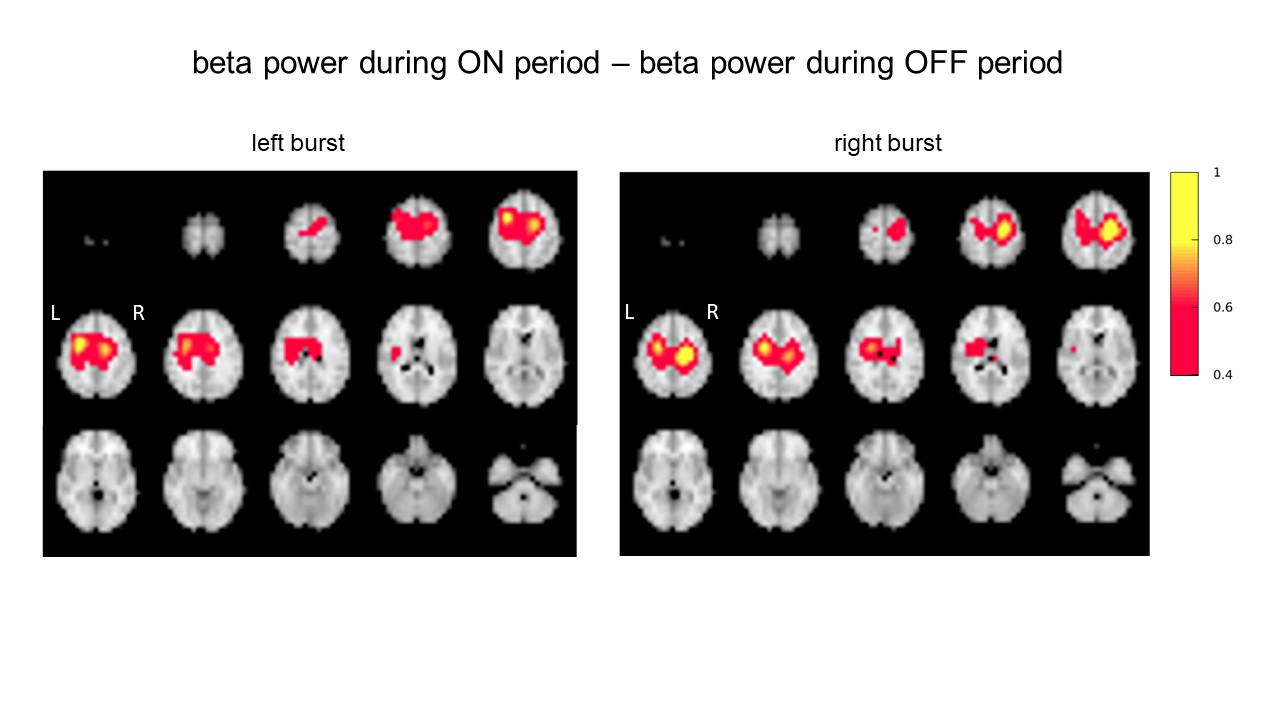


**Figure S10.** **Beta power difference between ON and OFF periods in source space.** We reconstructed the sources of our MEG signals and locked the source signal to the times of beta ON and OFF periods, as identified in the sensor signal. We extracted a 200 ms time-window around the beta ON and OFF periods, calculated the average beta power in this window and subtracted beta power in ON vs OFF periods per voxel and per participant. This was done separately for ON and OFF periods identified on the left and right MEG channels (left and right subplots, respectively). The participant-averaged beta power difference between ON and OFF periods is shown.


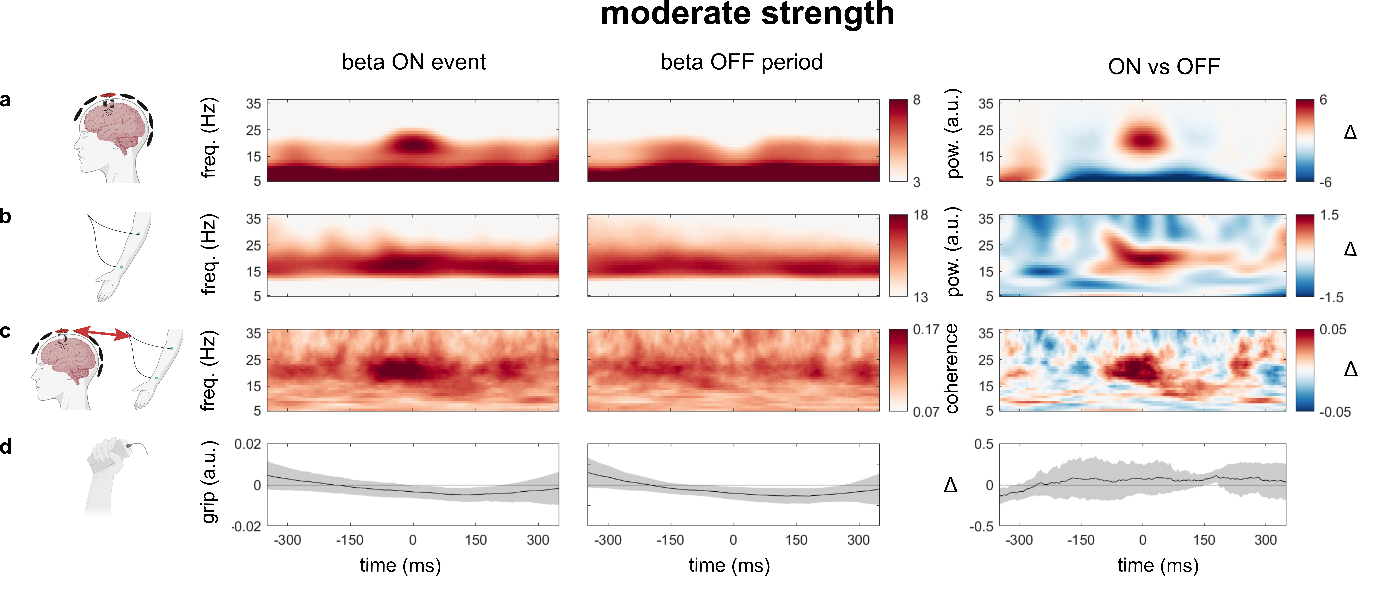


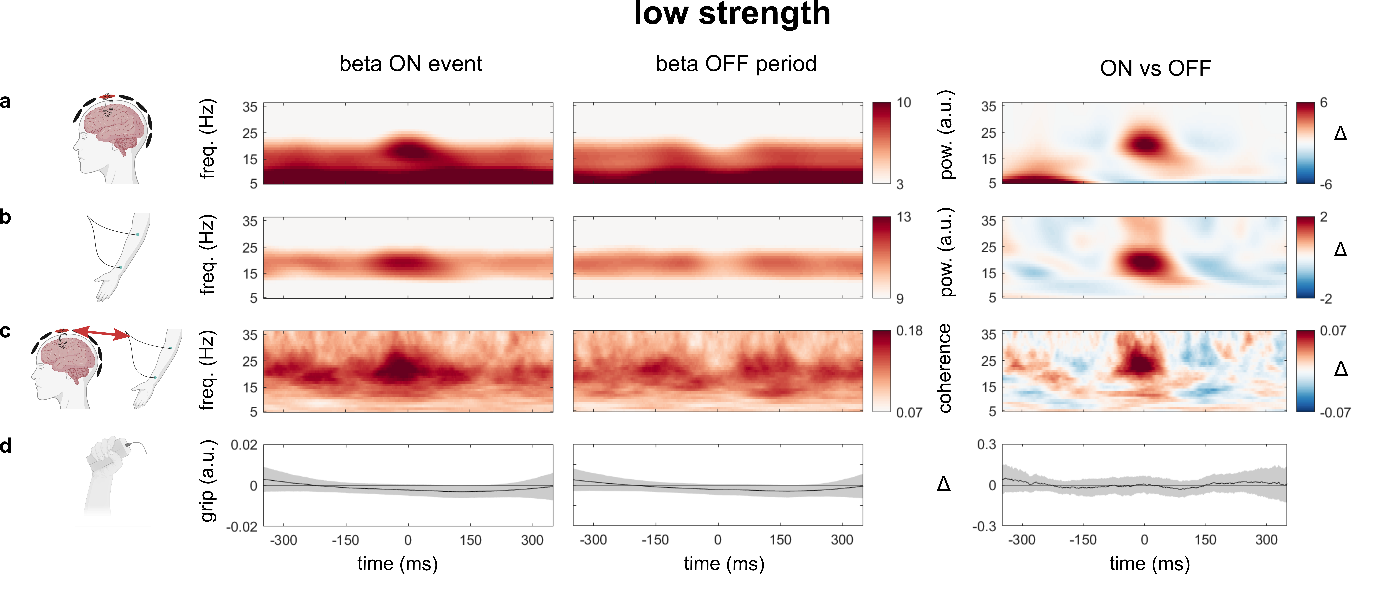


**Figure S11. Data aligned to beta ON and OFF periods in the brain reveal transient beta events in both strength conditions: low and moderate.** Beta ON events and OFF periods were identified using Empirical Mode Decomposition (EMD) in the selected MEG channels during the ~1-3s period of sustained gripping separately for low and moderate strength conditions. After ON and OFF period detection, data were aligned and averaged across all trials and all participants (N=18). Columns from left to right represent our four signals of interest (**a-d**) aligned to the central point of a beta ON event (left), a beta OFF period (middle) and the difference between them (right). **a**) MEG time-frequency spectrum, **b**) EMG time-frequency spectrum, **c**) CMC time-frequency spectrum and **d**) gripper signal. Shading indicates 95% confidence intervals. Black outlines in the right time-frequency maps indicate significant clusters.


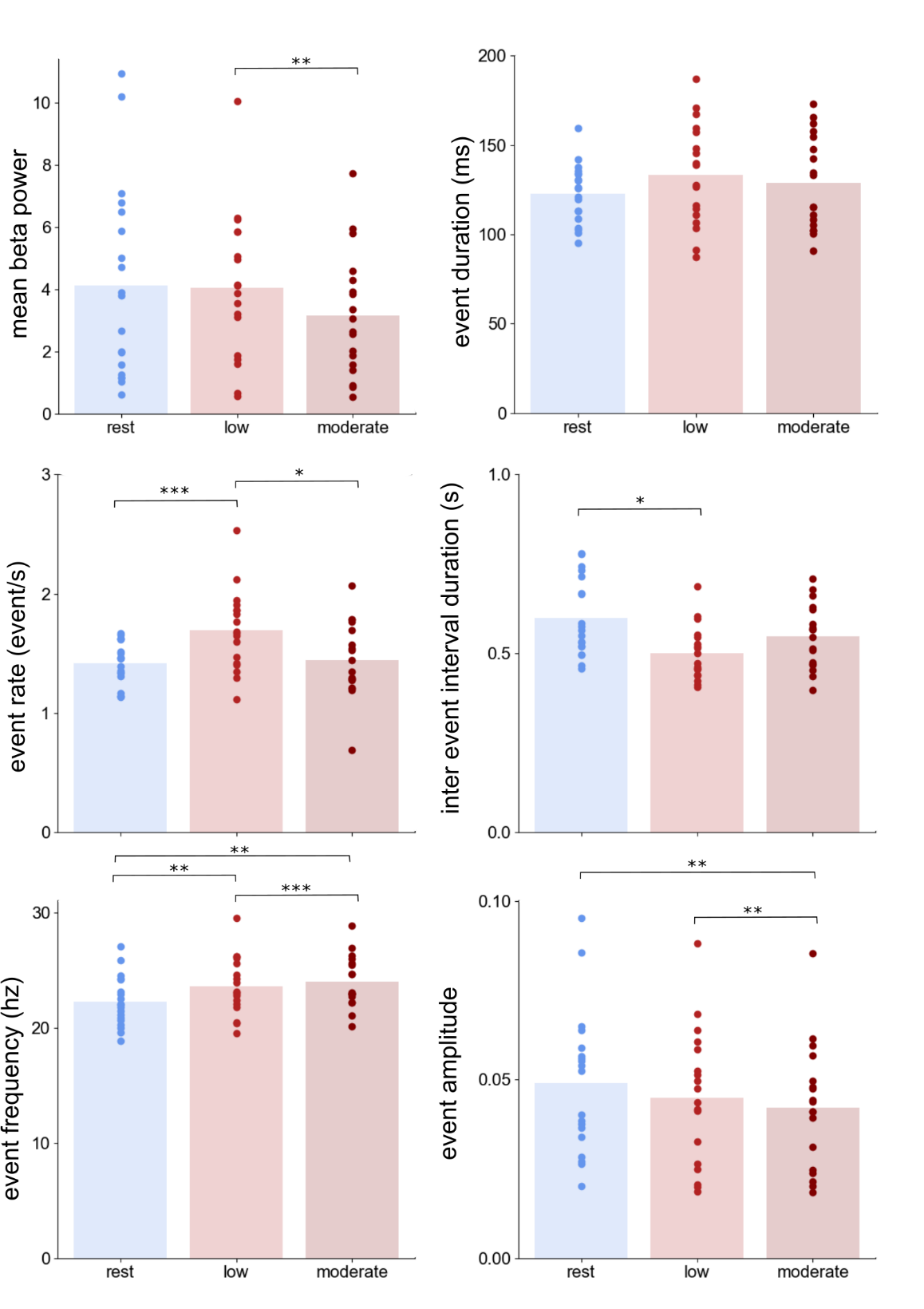


**Figure S12. Beta features (mean beta power, event duration, event rate, inter event interval duration, event frequency, and event amplitude) in the same participants during rest, low strength gripping and moderate strength gripping.** The average beta power during sustained gripping was calculated per participant (mean beta power). Beta event rate was calculated as number of events per second, the durations of the intervals between beta events were calculated in seconds and the mean frequency and amplitude of beta events were also quantified. Paired t-tests were used to compare the different conditions. We found differences in mean beta power between the low and moderate strength conditions (t_17_ = 3.35, p < 0.01). Statistically significant differences in event rate were found between rest and low strength gripping (t_17_ = 4.86, p < 0.001) and low and high strength (t_17_ = 2.38, p < 0.05). Differences in inter event interval durations were found between rest and low strength gripping (t_17_ = 2.41, p < 0.05). Additionally, mean frequency of beta events were different between rest and low gripping (t_17_ = 2.97, p < 0.01), low and moderate gripping (t_17_ = 4.2, p < 0.001) and rest and moderate gripping (t_17_ = -2.92, p < 0.01). Finally, differences in mean event amplitude were found between rest and moderate strength gripping (t_17_ = -3.27, p < 0.01) as well as low and moderate strength gripping (t_17_ = 2.93, p < 0.01).
